# Diagnostic prediction of neonatal hyperbilirubinemia combined with germinal matrix-intraventricular hemorrhage based on cranial ultrasound hemodynamics: a retrospective case-control study

**DOI:** 10.3389/fmed.2025.1606892

**Published:** 2025-09-11

**Authors:** Zhaolan Ye, Xiang Chen, Daoming Wu, Jiantao Lin, Lihua Zhang

**Affiliations:** ^1^Department of Ultrasound, Fujian Provincial Hospital, Fuzhou, Fujian, China; ^2^Department of Ultrasound, Fuzhou University Affiliated Provincial Hospital, Fuzhou, Fujian, China; ^3^Shengli Clinical Medical College of Fujian Medical University, Fuzhou, Fujian, China

**Keywords:** cranial ultrasound hemodynamics, germinal matrix-intraventricular hemorrhage, neonatal hyperbilirubinemia, nomogram, prediction

## Abstract

**Objective:**

To study the diagnostic prediction of cranial ultrasound hemodynamics in children with neonatal hyperbilirubinemia combined with germinal matrix-intraventricular hemorrhage (GMH-IVH).

**Methods:**

We included 148 hyperbilirubinemic neonates who underwent cranial ultrasound to obtain hemodynamic parameter indexes, and constructed a nomogram visual prediction model through unifactorial and multifactorial analyses to study the role of cranial ultrasound hemodynamic parameters in the diagnostic prediction of neonatal hyperbilirubinemia combined with GMH-IVH.

**Results:**

A total of 148 patients eligible for enrollment were included in this study, of which 40 children developed GMH-IVH, with an incidence rate of 27.03%. Multifactorial logistic stepwise regression analysis showed that mothers suffering from gestational hypertension, total bilirubin ≥15 mg/dl, anterior cerebral artery third day to first day resistance index ratio of ≥1, and middle cerebral artery third day to first day resistance index ratio of ≥1 were the independent risk factors for the development of GMH-IVH in neonatal hyperbilirubinemic infants (*P* < 0.05); and ROC analysis showed that the area under the ROC curve (AUC) of the prediction model was 0.821 (95% CI: 0.746–0.897, *P* < 0.001), indicating good predictive efficacy of the model (discrimination), and the Hosmer-Lemeshow test (χ^2^ = 7.779, *P* = 0.255) and the calibration curve showed that the model had a good goodness-of-fit (calibration). The predictive model was visualized by plotting nomogram.

**Conclusion:**

Craniocerebral ultrasound hemodynamics-related parameters combined with clinical features to construct a predictive model for early and effective prediction of the occurrence and prognosis of GMH-IVH in neonates with hyperbilirubinemia.

## 1 Introduction

Germinal matrix-intraventricular hemorrhage (GMH-IVH) is a type of neonatal craniocerebral hemorrhage associated with the embryonic germinal matrix layer, which can cause complications such as periventricular hemorrhagic infarcts, softening of cerebral white matter, and posthemorrhagic hydrocephalus, and it is a major cause of neonatal death and poor prognosis for survivors (1). Some studies have shown that even low-grade hemorrhage can still affect the intellectual development of the child, and in severe cases, even cerebral palsy (2–4). And neonatal hyperbilirubinemia is a common clinical metabolic disease, which is caused by the production of bilirubin exceeding the clearance of hepatic and intestinal bilirubin, and in severe cases, it can damage the central nervous system, cause bilirubin encephalopathy, and affect the prognosis (5–7). In recent years, the incidence of combined GMH-IVH in children with hyperbilirubinemia has been found to increase year by year. For children with hyperbilirubinemia, the occurrence of intracranial hemorrhage will inevitably aggravate the damage to the central nervous system of the children and affect the maturation of the nervous system, so how to prevent the occurrence of GMH-IVH in such children has become a proposition worth exploring. At present, most domestic and foreign studies on neonatal GMH-IVH are limited to the diagnosis after the occurrence of GMH-IVH, and there are few reports on GMH-IVH in children with hyperbilirubinemia. Therefore, based on cranial color ultrasound technology, this paper will construct a model based on the collection of cranial color Doppler hemodynamic parameters, clinical laboratory indicators and other related data from newborns with hyperbilirubinemia to predict the risk of GMH-IVH in these patients, and provide a reference basis for early prevention and intervention against GMH-IVH in newborns with hyperbilirubinemia in the clinic.

## 2 Materials and methods

### 2.1 Study subjects and groups

This was a retrospective case-control study that included a total of 148 hyperbilirubinemic neonates from the neonatal intensive care unit (NICU) of Fujian Provincial Hospital (Fuzhou, China) between January 2021 and June 2023. All participants met the enrollment criteria and were evaluated for the occurrence of GMH-IVH by cranial ultrasound examination on days 1, 3, and 7 after hospitalization. Relevant hemodynamic parameters were recorded during these examinations. Based on the ultrasound findings on day 7, participants were categorized into GMH-IVH and Non-GMH-IVH groups. Inclusion criteria: ① Meet the diagnostic criteria of neonatal hyperbilirubinemia; ② Admitted to our NICU; ③ GMH-IVH was not detected in the craniocerebral ultrasound results of day 1 and day 3. Exclusion criteria: ① Other intracranial hemorrhage (including intraventricular hemorrhage, choroid plexus hemorrhage, parenchymal hemorrhage, cerebellar hemorrhage, etc.); ② GMH-IVH due to other etiologies (e.g., sepsis, perinatal asphyxia, mechanical ventilation > 24 h, vasoactive drug exposure, or major congenital heart defects); ③ children who were unable to cooperate with the craniocerebral ultrasound examination; ④ children with incomplete clinical data. Informed consent was obtained from the guardians of all children during the cranial ultrasound examination. This study was performed in line with the principles of the Declaration of Helsinki. Approval was granted by the Clinical Research Ethics Committee of Fujian Provincial Hospital (K2024-12-042). Informed written consent was obtained from all participants.

### 2.2 Instruments and methods

#### 2.2.1 Craniocerebral ultrasound

A Kaili color Doppler diagnostic machine with a C6-13 small microconvex probe was used, and all children were examined three times after admission, on days 1, 3, and 7. The children were placed in the supine position while sleeping or resting, the probe and the operator’s hands were cleaned and disinfected, and the sagittal and coronal sections were continuously and dynamically scanned with fontanel and temporal fontanel as the transillumination windows, and the size of the hemorrhage and the condition of each cerebral ventricle were recorded; Peak systolic flow velocity (PSV), resistance index (RI), pulsatility index (PI) were measured in anterior cerebral artery (ACA) bilaterally; PSV, RI, PI in middle cerebral artery (MCA) bilaterally. The mean values of each parameter of bilateral ACA and MCA were calculated. All cranial ultrasound examinations were performed by the same senior sonographer with extensive work experience.

#### 2.2.2 Serum total bilirubin measurement

2 ml of fasting venous blood was collected from all children in the early morning before the first craniocerebral ultrasound was performed, and serum total bilirubin levels were measured by vanadate oxidation method using a fully automated biochemistry analyzer (Roche cobas c 702), which was done by the laboratory department of our hospital.

### 2.3 Relevant definitions

#### 2.3.1 Diagnostic criteria of GMH-IVH ultrasound

The echoes of the bleeding site are slightly enhanced in the early stage, with the contraction of the blood clot, the echoes are gradually enhanced, and when the bleeding is stable, a well-defined strong echogenic mass is formed, and thereafter the strong echogenic mass disappears gradually with the absorption of the bleeding (1).

#### 2.3.2 Clinical diagnostic criteria of hyperbilirubinemia

All enrolled neonates were clinically diagnosed with hyperbilirubinemia. The neonatal hourly bilirubin column chart produced by Bhutani et al. (8) or the phototherapy reference curve recommended by the American Academy of Pediatrics was used as a reference for diagnosis or intervention criteria. Hyperbilirubinemia was defined when the bilirubin level exceeded the 95th percentile.

### 2.4 Baseline information and study variables

General clinical information was collected including age of the child, sex, birth weight, whether the child was born prematurely, whether the child was born by cesarean section, and history of total bilirubin mother’s illness (thalassemia, thyroid dysfunction, gestational hypertension, gestational hyperglycemia).

Craniocerebral color ultrasound hemodynamic parameters were collected: ① the ratio of PSV of ACA on the third day to that of the first day (ACA_PSVd3/PSVd1); ② the ratio of RI of ACA on the third day to that of the first day (ACA_RId3/RId1); ③ the ratio of PI of ACA on the third day to that of the first day (ACA_PId3/PId1); ④ the ratio of PSV of MCA on the third day to the first day (MCA_PSVd3/PSVd1); ⑤ the ratio of the RI of the MCA on the third day to the first day (MCA_RId3/RId1); ⑥ the ratio of the PI of the MCA on the third day to the first day (MCA_PId3/PId1).

### 2.5 Statistical analysis

Measurement information conforming to normal distribution was expressed as mean ± standard deviation and independent samples *t*-test was used to compare the differences between groups; non-normally distributed measurements were expressed as median and interquartile range (IQR), and Mann-Whitney U test was used to compare the differences between groups; counting information was expressed as percentage, and χ^2^ test was used to compare the differences between groups; and the risk factors were analyzed by Logistic Stepwise Regression Analysis Risk factors were screened, and the final model was used to construct a nomogram predicting the occurrence of GMH-IVH. Statistical significance was defined as *P* < 0.05, and all analyses were performed using R software version 4.2.3 and SPSS software version 25.0.

## 3 Results

### 3.1 Description of the study population and comparison of general information

A total of 148 children were included in this study, of which 40 were in the GMH-IVH group, with an incidence rate of 27.0% (40/148). The median age of the children was 3 days, and the proportion of males was 50.7% (75/148); comparison of the data between the two groups showed that the proportion of children aged ≥3 days, with a birth weight ≤2,500 g, whose mothers suffered from gestational hypertension, with ACA_PSVd3/PSVd1 ≥ 1, and with MCA_PSVd3/PSVd1 ≥ 1, was higher in the GMH-IVH group (*P* < 0.05), preterm, total bilirubin ≥ 15 mg/dl, MCA_PId3/PId1 ≥ 1 proportionally higher (*P* < 0.01), ACA_RId3/RId1 ≥ 1, ACA_PId3/PId1, MCA_RId3/RId1 ≥ 1 proportionately higher (*P* < 0.001), and the difference of the remaining information comparison was not statistically significant (all *P* > 0.05) (Table 1).

**TABLE 1:** Baseline craniocerebral ultrasound hemodynamic and clinical characteristics of the study population.

Variable	All (*n* = 148)	GMH-IVH (*n* = 40)	Non-GMH-IVH (*n* = 108)	χ^2^/t/Z	*P*
Age (days)	3 (2, 3)	3 (3, 3)	3 (2, 3)	−1.219	0.223
Age ≥ 3 days, *N* (%)	105 (70.9%)	34 (85.0%)	71 (65.7%)	5.253	0.022*
Male, *N* (%)	75 (50.7%)	22 (55.0%)	53 (49.1%)	0.41	0.522
Birth weight ≤ 2500 g, *N* (%)	43 (29.1%)	17 (42.5%)	26 (24.1%)	4.808	0.028*
Premature birth, *N* (%)	56 (37.8%)	23 (57.5%)	33 (30.6%)	9.01	0.003**
Cesarean section child, *N* (%)	18 (12.2%)	6 (15.0%)	12 (11.1%)	0.413	0.520
Mother with thalassemia, *N* (%)	14 (9.5%)	5 (12.5%)	9 (8.3%)	0.592	0.442
Mother with thyroid dysfunction, *N* (%)	15 (10.1%)	6 (15.0%)	9 (8.3%)	1.379	0.240
Mothers with gestational hypertension, *N* (%)	16 (10.8%)	8 (20.0%)	8 (5.4%)	4.8	0.028*
Mothers with gestational hyperglycemia, *N* (%)	14 (9.5%)	5 (3.4)	9 (6.1%)	0.592	0.442
TBIL (mg/dl)	15.07 (12.58, 16.68)	15.46 (14.34, 17.12)	14.23 (12.25, 16.60)	−1.736	0.083
TBIL ≥ 15 mg/dl, *N* (%)	75 (50.7%)	29 (72.5%)	46 (42.6%)	10.445	0.001**
Cerebral color Doppler hemodynamic parameters, *N* (%)					
ACA_PSVd3/PSVd1 ≥ 1, *N* (%)	45 (30.4%)	18 (45.0%)	27 (25.0%)	5.518	0.019*
ACA_RId3/RId1 ≥ 1, *N* (%)	65 (43.9%)	28 (70.0%)	37 (34.3%)	15.138	<0.001***
ACA_PId3/PId1 ≥ 1, *N* (%)	60 (40.5%)	26 (65.0%)	34 (31.5%)	13.604	<0.001***
MCA_PSVd3/PSVd1 ≥ 1, *N* (%)	80 (54.1%)	27 (67.5%)	53 (49.1%)	3.99	0.046*
MCA_RId3/RId1 ≥ 1, *N* (%)	48 (32.4%)	23 (57.5%)	25 (23.1%)	15.718	<0.001***
MCA_PId3/PId1 ≥ 1, *N* (%)	56 (37.8%)	24 (60.0%)	32 (29.6%)	11.446	0.001**

The continuous data are expressed as the mean ± SD (normally distributed) or median and interquartile range (non-normally distributed). The count data are expressed as numbers (percentages). ACA, anterior cerebral artery; MCA, middle cerebral artery; PSV, peak systolic flow velocity; RI, resistance index; PI, pulsatility index; TBIL, total bilirubin; ACA_PSVd3/PSVd1, the ratio of PSV of ACA on the third day to that of the first day; ACA_RId3/RId1, the ratio of RI of ACA on the third day to that of the first day; ACA_PId3/PId1, the ratio of PI of ACA on the third day to that of the first day; MCA_PSVd3/PSVd1, the ratio of PSV of MCA on the third day to the first day; MCA_RId3/RId1, the ratio of the RI of the MCA on the third day to the first day; MCA_PId3/PId1, the ratio of the PI of the MCA on the third day to the first day. The Student’s *t*-test is used to compare normally distributed continuous variables, while the Mann–Whitney U test is used to compare non-normally distributed continuous variables. The chi-squared test is used to compare categorical variables.

*P < 0.05, **P < 0.01, ***P < 0.001.

### 3.2 Univariate analysis of risk factors associated with the development of GMH-IVH in neonates with hyperbilirubinemia

The results of univariate analysis showed that age ≥3 days, birth weight ≤ 2500 g, prematurity, mother suffering from gestational hypertension, total bilirubin ≥ 15 mg/dl, ACA_PSVd3/PSVd1 ≥ 1, ACA_RId3/RId1 ≥ 1, ACA_PId3/PId1 ≥ 1, MCA_PSVd3/PSVd1 ≥ 1, MCA_RId3/RId1 ≥ 1, and MCA_PId3/PId1 ≥ 1 were risk factors for the development of GMH-IVH in neonatal hyperbilirubinemia (*P* < 0.05) (Table 2).

**TABLE 2:** Univariate analysis of risk factors for the occurrence of GMH-IVH in neonates with hyperbilirubinemia.

Variable	Beta	OR	CI	*P*
Age	0.114	1.12	0.80–1.58	0.511
Age ≥ 3 days	1.097	3	1.15–7.78	0.024*
Male	0.257	1.29	0.62–2.68	0.490
Birth weight ≤ 2500 g	0.834	2.3	1.07–4.96	0.033*
Premature birth	1.154	3.17	1.50–6.72	0.003**
Cesarean section child	0.334	1.4	0.49–4.01	0.535
Mother with thalassemia	0.442	1.56	0.49–4.96	0.455
Mother with thyroid dysfunction	0.653	1.92	0.64–5.79	0.246
Mothers with gestational hypertension	1.129	3.09	1.07–8.92	0.036*
Mothers with gestational hyperglycemia	0.442	1.56	0.49–4.96	0.455
ACA_PSVd3/PSVd1 ≥ 1	0.886	2.42	1.13–5.19	0.022*
ACA_RId3/RId1 ≥ 1	1.526	4.6	2.10–10.1	<0.001***
ACA_PId3/PId1 ≥ 1	1.427	4.16	1.93–8.98	<0.001***
MCA_PSVd3/PSVd1 ≥ 1	0.787	2.2	1.02–4.71	0.043*
MCA_RId3/RId1 ≥ 1	1.543	4.68	2.16–10.15	<0.001***
MCA_PId3/PId1 ≥ 1	1.302	3.68	1.72–7.85	0.001**
TBIL (mg/dl)	0.019	1.02	0.93–1.11	0.677
TBIL ≥ 15 mg/dl	1.29	3.63	1.64–8.03	0.001**

ACA, anterior cerebral artery; MCA, middle cerebral artery; PSV, peak systolic flow velocity; RI, resistance index; PI, pulsatility index; TBIL, total bilirubin; ACA_PSVd3/PSVd1, the ratio of PSV of ACA on the third day to that of the first day; ACA_RId3/RId1, the ratio of RI of ACA on the third day to that of the first day; ACA_PId3/PId1, the ratio of PI of ACA on the third day to that of the first day; MCA_PSVd3/PSVd1, the ratio of PSV of MCA on the third day to the first day; MCA_RId3/RId1, the ratio of the RI of the MCA on the third day to the first day; MCA_PId3/PId1, the ratio of the PI of the MCA on the third day to the first day. OR, odds ratio; CI, confidence interval.

*P < 0.05, **P < 0.01, ***P < 0.001.

### 3.3 Multifactorial analysis of the risk and visualization of GMH-IVH in neonates with hyperbilirubinemia

**FIGURE 1:**
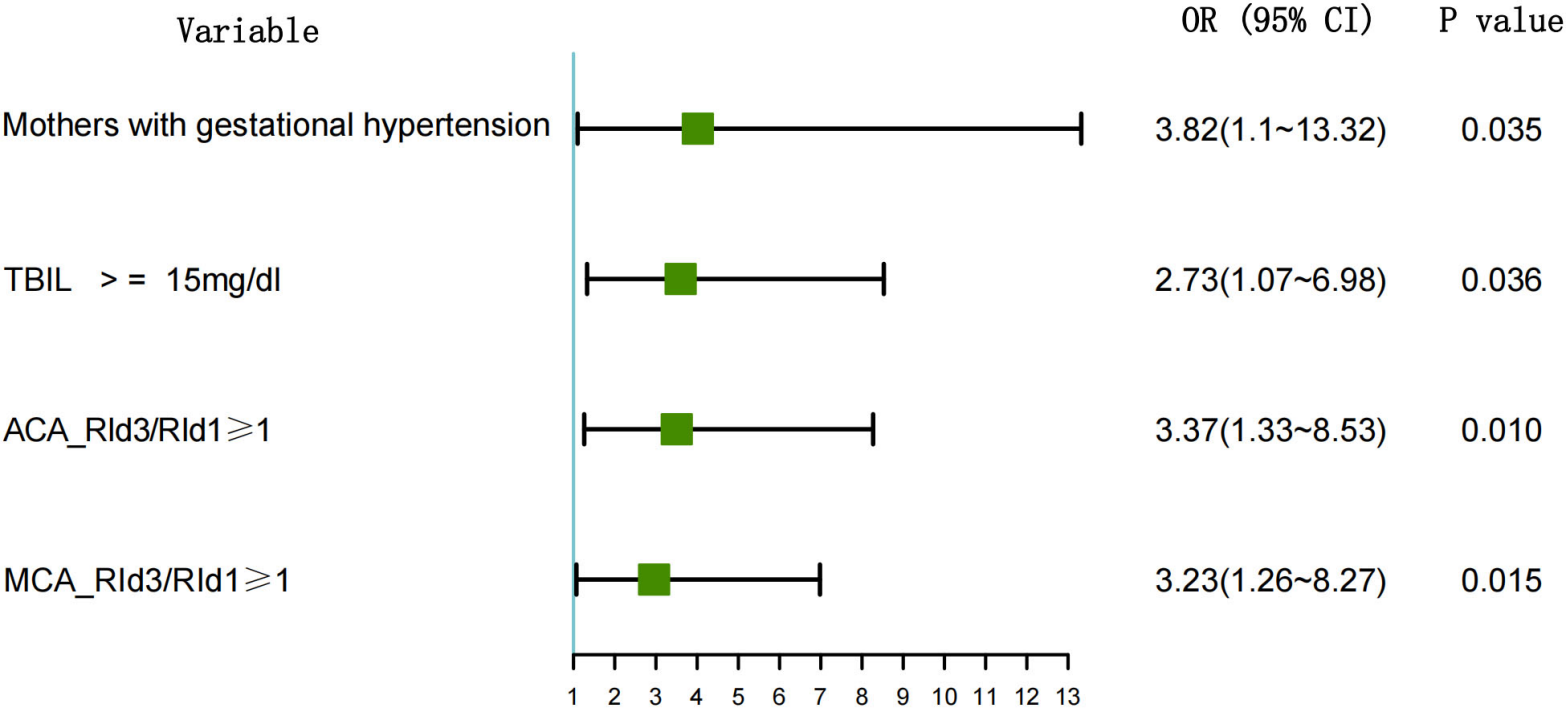
Forest plot of multivariate analysis for GMH-IVH.

Variables that were significant in one-way analysis (*P* < 0.05) were included in multifactorial logistic stepwise regression analysis, and the results showed that mothers with gestational hypertension, total bilirubin ≥ 15 mg/dl, ACA_RId3/RId1 ≥ 1, and MCA_RId3/RId1 were the independent risk factors for the development of GMH-IVH in neonates with hyperbilirubinemia (*P* < 0.05) (Table 3). For hyperbilirubinemic neonatal infants, if the mother suffered from gestational hypertension, the total bilirubin of the child was ≥15 mg/dl, and the hemodynamic parameters of craniocerebral ultrasound suggested that ACA_RId3/RId1 ≥ 1 and MCA_RId3/RId11 ≥ 1, there was a high risk of developing GMH-IVH, and the ORs and confidence intervals were respectively, 3.82 (1.1–13.32), 2.73 (1.07–6.98), 3.37 (1.33–8.53), and 3.23 (1.26–8.27), respectively (Figure 1).

**TABLE 3:** Multivariate analysis of risk factors for the occurrence of GMH-IVH in neonates with hyperbilirubinemia.

Variable	Beta	OR	CI	*P*
Mothers with gestational hypertension	1.341	3.82	1.1–13.32	0.035*
TBIL ≥ 15 mg/dl	1.004	2.73	1.07–6.98	0.036*
ACA_RId3/RId1 ≥ 1	1.216	3.37	1.33–8.53	0.01*
ACA_PId3/PId1 ≥ 1	0.7	2.01	0.79–5.1	0.14
MCA_RId3/RId1 ≥ 1	1.171	3.23	1.26–8.27	0.015*
MCA_PId3/PId1 ≥ 1	0.751	2.12	0.83–5.37	0.114

ACA, anterior cerebral artery; MCA, middle cerebral artery; RI, resistance index; PI, pulsatility index; TBIL, total bilirubin; ACA_RId3/RId1, the ratio of RI of ACA on the third day to that of the first day; ACA_PId3/PId1, the ratio of PI of ACA on the third day to that of the first day; MCA_RId3/RId1, the ratio of the RI of the MCA on the third day to the first day; MCA_PId3/PId1, the ratio of the PI of the MCA on the third day to the first day. OR, odds ratio; CI, confidence interval.

*P < 0.05.

### 3.4 Evaluation of model prediction efficacy and goodness-of-fit

**FIGURE 2:**
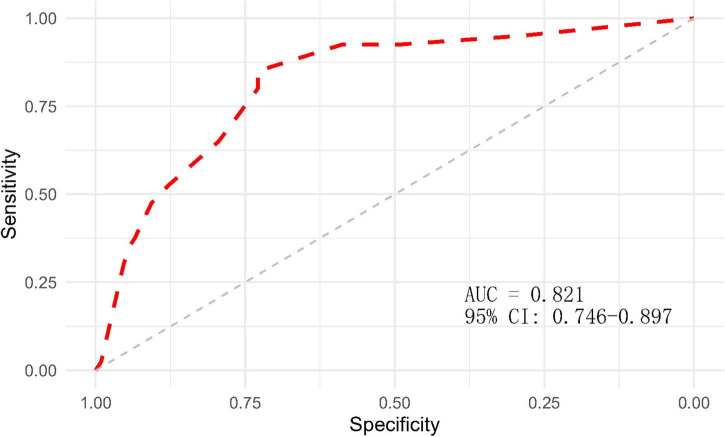
ROC curve of the GMH-IVH prediction model.

**FIGURE 3:**
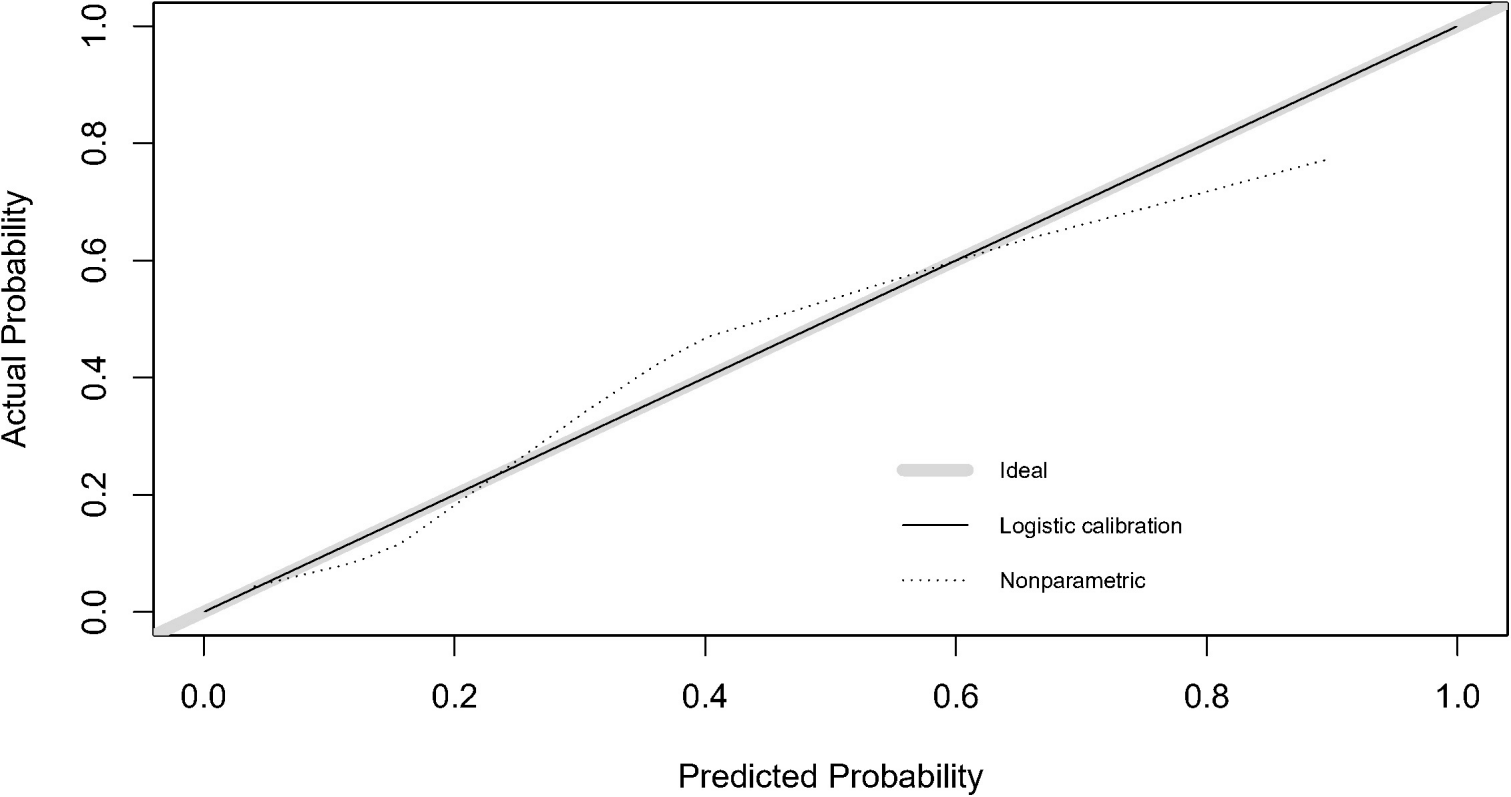
Calibration curve of the GMH-IVH prediction model.

After completing the above multifactor logistic stepwise regression analysis modeling, the predictive efficacy (differentiation) of the model was further evaluated by the area under the ROC curve (AUC), and the results showed that the AUC of the ROC of the model was 0.821 (95% CI: 0.746–0.897, *P* < 0.001) (Figure 2), indicating that the model has a good differentiation, and the predictive efficacy was good. The goodness-of-fit of the model was tested by the Hosmer-Lemeshow test (HL: χ^2^ = 7.779, *P* = 0.255) and calibration curve (Figure 3), and the results indicated that the model had good goodness-of-fit (calibration). The evaluation results indicated that the model has good clinical application for predicting the risk of GMH-IVH in children with hyperbilirubinemia.

### 3.5 Plotting nomogram

**FIGURE 4:**
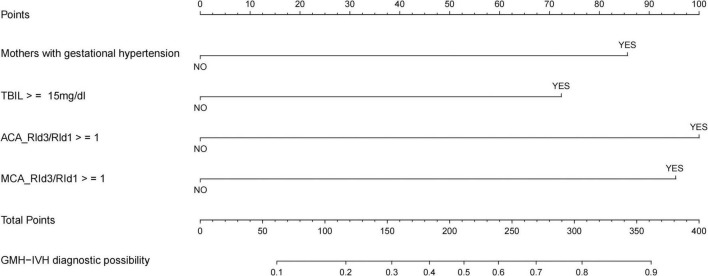
Nomogram for the diagnostic probability of GMH-IVH.

Based on the multifactor logistic stepwise regression outcome model, a nomogram was drawn to visualize the outcome model (Figure 4).

## 4 Discussion

Germinal matrix-intraventricular hemorrhage is the most common neurological lesion in the neonatal period, which has a high mortality rate, and children who survive in the long term are often accompanied by different degrees and types of neurological sequelae, such as motor deficits, visual and auditory deficits, and backwardness in intellectual development (9). Hyperbilirubinemia, as one of the major diseases admitted to NICU, has been clinically found in recent years that the incidence of such children with GMH-IVH is increasing year by year, which not only increases the chance of neurological damage and prolongs the average length of hospitalization, but also increases the family’s financial burden and health insurance expenditure, which affects the children themselves, their families, hospitals, and the society to varying degrees. Cranial ultrasound is currently recognized as the imaging diagnosis of choice for screening GMH-IVH due to its advantages of safety and non-radiation, bedside examination, and high resolution of the central part of the brain (4, 10). At present, a number of studies based on cranial ultrasound pointed out that the cerebral hemodynamics of preterm infants with intracranial hemorrhage will produce a series of changes, such as: Li Junling (11) and others believe that the ACA and MCA flow velocity increases within 72 h after birth in children with intracranial hemorrhage, and that the out 24-h average cerebral blood flow velocity correlates with the mean arterial blood pressure; Liu Dengli (12) and other experts’ findings show that children who develop intracranial hemorrhage have an increased risk of intracranial vasoconstriction in the first 24 h of life. The peak systolic blood flow velocity, mean blood flow velocity and diastolic blood flow velocity of intracranial vessels were significantly elevated within 24 h, and the intracranial blood perfusion situation had a certain predictive effect on intracranial hemorrhage in newborns. However, the occurrence of GMH-IVH in children with predicted hyperbilirubinemia has been rarely reported. In view of this, this study synthesized the clinical history, test indexes, and dynamically observed craniocerebral ultrasound hemodynamic comparison parameters to construct an early prediction model for the occurrence of GMH-IVH in infants with hyperbilirubinemia, and to provide assistance for the establishment of clinical early warning and intervention mechanisms.

In this study, we dynamically observed the changes of cranial hemodynamic parameters related to cranial hemodynamics in children with hyperbilirubinemia through the clinic, and used six indexes, ACA_PSVd3/PSVd1, ACA_RId3/RId1, ACA_PId3/PId1, MCA_PSVd3/PSVd1, MCA_RId3/RId1, and MCA_PId3/PId1, as the observational variables reflecting the changes in cranial hemodynamics on the third day versus the first day. The results showed that ACA_RId3/RId1 ≥ 1 and MCA_RId3/RId1 ≥ 1 were independent risk factors for the development of GMH-IVH in children with hyperbilirubinemia, and a higher proportion of patients with ACA and MCA day 1 versus day 3 RI ratios ≥ 1 were found in the GMH-IVH group compared with the Non-GMH-IVH group, indicating that although no definite hemorrhagic lesions were detected in the children on the craniocerebral ultrasound examination, the proportion of patients with ACA and MCA day 1 versus day 3 RI ratio ≥ 1 was higher in this group. No clear hemorrhagic foci have been found, but the intracranial vascular RI values showed a trend of gradual increase over time, which may be due to the intracranial hypoperfusion in hyperbilirubinemic children (13, 14), cerebral vasospasm, slowing down of blood flow, and intracranial hypoperfusion will further aggravate the symptom of jaundice in the children. Therefore, regular dynamic observation of the RI of ACA and MCA in children with hyperbilirubinemia can reflect the distribution of intracranial blood perfusion and the resistance of blood vessels to a certain extent, which has positive clinical significance in predicting GMH-IVH caused by intracranial vascular rupture.

In addition to this, in the present study, two indicators, the mother suffering from gestational hypertension and total bilirubin ≥ 15 mg/dl, were also found to be independent risk factors for the development of GMH-IVH in neonates with hyperbilirubinemia. It has been shown that gestational hypertension may lead to constriction and narrowing of the small spiral arteries, which affects oxygen exchange in the placenta, and increases the probability of central nervous disorders in newborns (15, 16), which in turn increases the probability of GMH-IVH. In the case of total bilirubin, our study showed that only when total bilirubin reaches or exceeds the level of 15 mg/dl, and bilirubin metabolism disorders are further exacerbated, does it have an impact on the development of GMH-IVH in newborns.

In this study, we constructed a model and plotted the nomogram for the occurrence of GMH-IVH in hyperbilirubinemic neonates by using independent risk factors screened by multifactorial logistic analysis (mother suffering from gestational hypertension, total bilirubin ≥ 15 mg/dl, ACA_RId3/RId1 ≥ 1, and MCA_RId3/RId1 ≥ 1). After modeling, the predictive efficacy of the model was evaluated by the area under the ROC curve, and the goodness of fit of the model was detected by the Hosmer-Lemeshow test (χ^2^ = 7.779, *P* = 0.255) and calibration curve. The model consists of three parts, namely, the variables of the prediction model, the corresponding scores of the variables, and the probability of occurrence of the predicted events. After completing the craniocerebral ultrasound hemodynamic examination on days 1 and 3 after the admission of a child with hyperbilirubinemia to the NICU, NICU medical staff can visualize the scores of the child’s various items in the predictive map, taking into account the clinical total bilirubin level and the mother’s history of gestational hypertension. This can be used to individually predict the risk of GMH-IVH in children with hyperbilirubinemia for early warning of high-risk groups and early intervention to prevent further progression of the disease.

In summary, the nomogram model constructed in this study can quantitatively assess and predict the risk of GMH-IVH in children with hyperbilirubinemia more accurately, and can provide a valuable auxiliary role for the early prediction and prevention of GMH-IVH in newborn children with hyperbilirubinemia. However, there are some limitations in the study. First, the single-center, retrospective design with a relatively small sample size may introduce selection and population bias, limiting the generalizability of our findings to broader, more diverse populations. And the absence of an external validation cohort further restricts the robustness of our model. Second, the short observation period precluded assessment of long-term neurodevelopmental outcomes, potentially missing cases of delayed-onset IVH. Furthermore, logistical constraints (e.g., follow-up compliance) prevented inclusion of extended hemodynamic monitoring data (e.g., days 14, 21, 28), leaving the model unvalidated in longer-term cohorts. Considering the limitations, future research will be conducted by expanding the sample size, incorporating multicenter prospective designs with broader demographic representation, and establishing a long-cycle follow-up observation cohort to improve the model’s robustness and generalizability over longer time horizons.

## Data Availability

The data analyzed in this study is subject to the following licenses/restrictions: Data are available on request due to restrictions such as privacy or ethical reasons. Requests to access these datasets should be directed to Z15859046516@aliyun.com.
